# Modelling the Effects of Future Climate Conditions on Oil Palm Microbial Diseases and Poor Oil Palm Development, Compared to the Effects on Other Crops, Provides Possible Solutions

**DOI:** 10.3390/microorganisms14071532

**Published:** 2026-07-14

**Authors:** Robert Russell Monteith Paterson

**Affiliations:** Centre of Biological Engineering, Gualtar Campus, University of Minho, 4710-057 Braga, Portugal; russell.paterson@deb.uminho.pt

**Keywords:** *Elaeis guineensis*, *Ganoderma boninense*, *Phytophthora palmivora*, *Fusarium oxysporum* f. sp. *ealanis*, soybeans, CLIMEX, Colombia, fungi, oomycete

## Abstract

Maintaining food systems in the face of climate change (CC) is a major concern. Palm oil is included in many commodities, and oil palms (OPs) will be adversely affected by an inclement future climate, which could cause OPs to experience increases in disease incidence, higher mortality and a decline in growth. Basal stem rot, bud rot and Fusarium wilt of the OP are considered in the present paper, as attempts to control these diseases have been unsuccessful. A new approach may be the replacement of compromised OPs with different crops better suited to the future climate and which may exhibit fewer diseases initially because of the “parasites lost” phenomenon. Maintaining a vegetable oil product is an important advantage of this approach. How CC will affect OPs has been determined previously by CLIMEX modelling. The modelling of a future suitable climate has also been carried out for soybeans, maize and the common bean using the same modelling parameters. This enables direct comparisons with the OP production in countries such as Colombia, Nigeria and Papua New Guinea. Limited data for rapeseed are also available. The results show that the suitability of future suitable climate for OPs was much reduced in these countries and that for soybeans remained beneficial. Under these conditions, soybeans may exhibit fewer diseases, as they would be an introduced and annual crop. Maize exhibited far fewer advantages, and the common bean and rapeseed showed none. Maize exhibited potential advantages in Nigeria until 2050. A novel method for adapting to the serious diseases, poor growth and mortality of OPs would be to grow soybeans in regions similar to those in which OPs currently grow, but for which modelling suggests that their health will become limited. Plans, which could be modified when real-time data become available, could be made for replacing OPs with soybeans. This current paper provides a novel method for mitigating the future diseases, poor growth and mortality of OPs whilst maintaining valuable oil production.

## 1. Introduction

Protecting food systems from climate change (CC) is essential. The earth is reaching climate tipping points, imposing long-lasting and irreversible effects leading to a hothouse earth trajectory [1], with negative consequences for growing crops. Temperatures may become sufficiently high to make the manual labour required in crop production impossible [2]; shifting tropical rainfall patterns and increased drought are also anticipated. The situation is worse than previously determined, as the 1.5 °C warming limit set by the Paris Agreement is currently estimated to be breached by ca. 2030, if the warming rate of the past 10 years continues [3], resulting in the possibility of dramatically negative consequences for crop production. If proven correct, these scenarios will detrimentally affect food systems, causing significant general concern.

Palm oil is very important and is employed frequently in the food industry. However, other oils may more often be included in supermarket products [4]. Palm oil is included in biodiesel, cosmetics and pharmaceuticals and is used commonly in domestic cooking. Malaysia and Indonesia produce the largest volumes of palm oil, although Colombia, Nigeria and Papua New Guinea (PNG) produce significant quantities. Interestingly, Nigeria manufactures amounts of global oil crops similar to the quantities produced in Malaysia [5].

Climate change could affect palm oil production detrimentally, causing increasing incidences of diseases such as basal stem rot (BSR), with large areas of producing countries becoming unable to grow OPs due to the inclement climate [6]. Palm oil production is particularly labour-intensive, making manufacturing much more difficult under CC conditions [2]. Procedures are required to ameliorate the effects of CC on OP production [6], thereby increasing the sustainability of the palm oil industry. Growing modified OPs to combat BSR in the future may not bring dramatic improvements in OP growth [7], and modified OPs produced to address CC may not significantly affect declines in palm oil yields [8]. An alternative would be to grow other crops suited to the future climate conditions, which may have low initial incidences of disease, because the new crop would be introduced into a novel environment [9,10], thereby providing an advantage over growing the heavily-infected OPs. Comparing equivalent future climate modelling of crops is a potential method to discover candidates for replacing OPs [6].

Negative impacts on canola (rapeseed) plants from CC in Canada and Europe were determined by using models ([11] and [12], respectively). The effects on sunflower plants were favourable in northern Europe, with large declines in southern and eastern Europe [13]. Crop yield scenarios under CC suggested losses for maize, soybeans and rice, with gains for wheat [14], indicating how CC can have widely varying effects on different crops. Finally, large areas, such as in the tropics, were expected to become unsuitable for soybeans under CC [15], although Southeast Asia maintained high suitability for these crops [6].

Nevertheless, crop growers may not respond to the negative effects of CC, leading to increased vulnerability of their crops, which can be considered as an inappropriate response (i.e., maladaptation). Coping strategies can be short-term, which are expensive and may comprise maladaptation if the situation does not improve. For the small-scale farmer, this may be expressed as the pawning of family assets to buy poor quality seeds. A suitable adaptation strategy may involve coping in the short term and then building upon this behaviour in the longer term, leading to positive outcomes based on learning [16].

One method for adapting to CC is to replace a susceptible product with one more suited to the novel climate, which may represent a difficult, but necessary, change. For example, farmers in Bangladesh rear ducks, rather than the traditional chickens, to cope with flooding because of CC. The chickens were drowned by the flooding, whereas the ducks could swim [17]. *Glycine soja* is the wild relative of the cultivated soybean, *G. max*, which is susceptible to various biotic and abiotic stresses. *G. soja* is climate-resilient and could be employed to replace *G. max* [18]. Cereal crops, which are susceptible to climate stress, are grown extensively in India, whereas millet offers a beneficial alternative because it is more resilient [19]. Perennial crops, such as OPs, have greater potential for future food security and environmental sustainability than do annual crops (e.g., soybeans) [20], which would suggest an advantage for OPs. However, the effects of the future climate on these crops are not adequately considered. Many influences, such as the global economy and dietary trends, determine how effective an adaptation strategy can be.

CLIMEX modelling has been used extensively to provide scenarios of the effect of CC on various species, often by generating climate maps [21]. The modelling of soybeans using CLIMEX [22] indicated that the climate would remain suitable in northern latitudes, with losses in tropical areas, although Southeast Asian regions retained high suitability [6]. The tropics exhibited a decrease in suitable climates for maize, with increases in a poleward direction [23]. Some of the modelling for these crops used the same parameters as those used for OPs, although they did not focus on the areas where OPs are grown commercially. The maps in individual papers can be readily assessed by the reader using visual inspection to determine how much suitable climate in a given region is available for a species to survive or grow [22,24,25]. A completely suitable region would represent 100% suitability, and a region which was unsuitable would represent 0% suitability, if quantification was required. The intermediate quantities can also be readily determined [6,23].

However, it is inconvenient to compare maps from different studies, even though these comparisons can be useful in determining which crops are suitable for a particular region. This was resolved in reference [6] by plotting the suitability determinations in graphs using straightforward comparisons. In a study that compared different crops using the same modelling parameters for Malaysia, Indonesia and Thailand, the future suitable climate for soybeans frequently covered a larger area than that for OPs [6]. There are clear benefits from growing OPs. It is a uniquely high-yielding and land-sparing crop and is associated with ongoing environmental and yield improvements [5]. Replacing OPs with other crops is not advantageous unless the climate deteriorates greatly from the present situation and/or diseases become severe. Maize and the common bean did not present such advantages in terms of future suitable climates [6]. In addition, an unsuitable climate existed until 2040 to 2059 for rapeseed in Malaysia and Thailand, according to CLIMEX programming [26], although different modelling parameters from those in [6] were employed. The equivalent data for Indonesia indicated that an approximate 20% suitability at the present time changed to 0% by 2040 to 2059 [26]. Hence, rapeseed would not be considered a suitable alternative to OP in these countries. Overall, OPs had better future climate suitability than maize, the common bean, and rapeseed and lower suitability than soybeans. There is a requirement to determine the equivalent situation in other palm oil producing countries where the impact and control of diseases in the future is also of crucial concern [27,28,29].

Fungal diseases and bud rot caused by *Phytophthora palmivora* are prevalent in OPs. *P. palmivora* is an oomycete [27], which is similar to a fungus, and occurs frequently in South America. BSR is a major problem especially in Southeast Asia and Papua New Guinea (PNG) and is caused by the fungus *Ganoderma boninense* [6]. The primary fungal disease of OPs in Africa is Fusarium wilt caused by *Fusarium oxysporum* f. sp. *elaeidis* [28]. These ailments have persisted for decades despite numerous attempts to control or eradicate them. They are projected to increase dramatically with CC under narrative modelling scenarios [6,27,28], threatening the sustainability of the OP industry; thus, successful management of the diseases is crucial.

One solution would be to replant highly diseased OP plantations with another crop which is not growing in the same region. The introduced crops are likely to have low levels of disease due to the “parasites lost” phenomenon, where introduced crops are initially susceptible to fewer pests and diseases than are the currently growing OPs [9,10]. As is indicated by modelling, this initial advantage may persist concerning the effects of the future climate on crop diseases [30,31]. Eliminating BSR is a goal which has long taxed researchers and palm oil producers [32]. Hence, introducing novel crops, such as soybeans, would be one procedure for overcoming OP diseases whilst still producing vegetable oil. By growing soybeans to replace OPs, the future climate effects on and diseases of OPs could be defeated [6].

The current paper compares the modelling of a future suitable climate for OPs to that for soybeans, maize and the common bean in Colombia, Nigeria and PNG. The fungal and fungal-like diseases of OPs are discussed using narrative models and are compared to the situation in which novel crops may be introduced. A new measure for OP mortality due to climate and disease is introduced. Rapeseed suitability is also discussed. This is the first time that (a) such a comparison has been made in these countries and (b) bud rot and Fusarium wilt have been considered. The proposal for replacing compromised OPs with soybeans [6] is reaffirmed, in some cases.

## 2. Materials and Methods

The future climate maps in reference [24] for 2015, 2050 and 2100 were used for OP in the present paper, from which the scenarios for Colombia, Nigeria and PNG were determined. Only the data for the CSIRO-Mk3.0 global climate model running SRES A2 were employed because these were the only parameters common to all the target papers for soybeans, maize and the common bean (references [22,23,25], respectively). The maps were magnified on a computer screen using the well-known standard magnification tool provided by personal computers. The percentages of suitable climates were assessed visually to provide percentage suitabilities for growing OPs, as determined by the colours of the maps [6,27,28]. The orientation of suitable climates was also determined within each country. The assessments were repeated numerous times throughout the preparation of the paper, which provided confirmation of the determinations.

The distribution model for OPs was developed using CLIMEX for Windows, Version 347 (Hearne Scientific Software Pty Ltd., Melbourne, Australia, 2007) under different climate scenarios, and climate data and CC scenarios were assessed using CliMond 10’gridded climate data. The potential future climate was characterised using A2 SRES scenarios, which are available from the CliMond dataset. The Global Biodiversity Information Facility was used for the fitting of CLIMEX parameters, and the information on the global distribution of OPs was used in parameter fitting. Southeast Asian distribution data were reserved for validation of the model. The OP distribution was determined by the Global Biodiversity Information Facility (GBIF) (http://www.gbif.org/, Occurrence search) (accessed on 9/12/2015) and additional literature on the species derived from CAB Direct (http://www.cabdirect.org/web/about.html, accessed 9 October 2015), forming the basis for the collection of data regarding the distribution of OPs. A mechanistic niche model using CLIMEX software supported ecological research incorporating the modelling of the potential distribution of species under differing climate scenarios and assumed that climate was the paramount determining factor for plant and poikilothermal animal distributions. The output from CLIMEX was categorized into areas, and these were also based on other studies conducted through CLIMEX.

The Ecoclimatic Index (EI), which is scaled from 0 to 100, was used to weight the degree of suitability in the maps. The establishment of OP suitability is only possible if EI > 0; 1–10 indicates marginal (syn. borderline) habitats, 10–20 is supportive of substantial populations, whilst > 20 is highly favourable for OP establishment. To provide a recognition of the indices, only 33% of the area on a map was determined as suitable if the relevant part showed marginal suitability. If a part of the map earned a substantial populations assessment, then 66% of the area was considered suitable. Finally, 100% of the area of the map was considered suitable if the map had a highly favourable assessment [6]. The percentages were based on the previously described delineation of the EI values and were considered more valid than results obtained by simply giving equal weight to various suitabilities, such as by combining highly suitable and suitable climate into one “suitable” category [33]. The combined average areas were designated as the weighted suitable climate (WSC). Disease was determined from inclement climate data in the three countries by adjusting published data [27,28,29], as employed in Table 1.

In addition, OP mortality was determined as a combination of death from disease and inclement climate. 

The climate maps provided for OPs [24] were compared with those featured in the other core papers used in the present study for soybeans [22], maize [23] and the common bean [25] using the same global climate model CSIRO-Mk3 and the A2 future climate scenarios used for OPs. The maps for maize, soybeans and the common bean were treated in the same manner as were the OP maps. The data for rapeseed were discussed separately for comparison [26].

The percentage areas for WSC, disease incidences and OP mortalities were plotted using Excel. The trend lines were inserted using the Excel tool to give an estimation of the rate of change of WSC. Values of the trend line were occasionally greater than 100%, which was a result of the mathematical extrapolation and did not represent a suitable climate value per se.

## 3. Results

**Figure 1:**
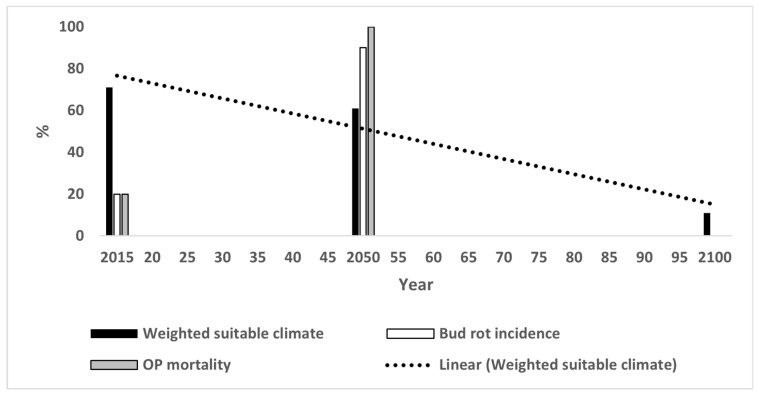
Weighted suitable climate, bud rot incidence and mortality of oil palms from inclement climate projected into the future for Colombia. The oil palm mortality was 100% in 2050.

The WSC values for OPs in Colombia were 71%, 61% and 11% for 2015, 2050 and 2100, respectively, with a trend of 80% to 18% from 2015 to 2100, respectively (Figure 1).

**Figure 2:**
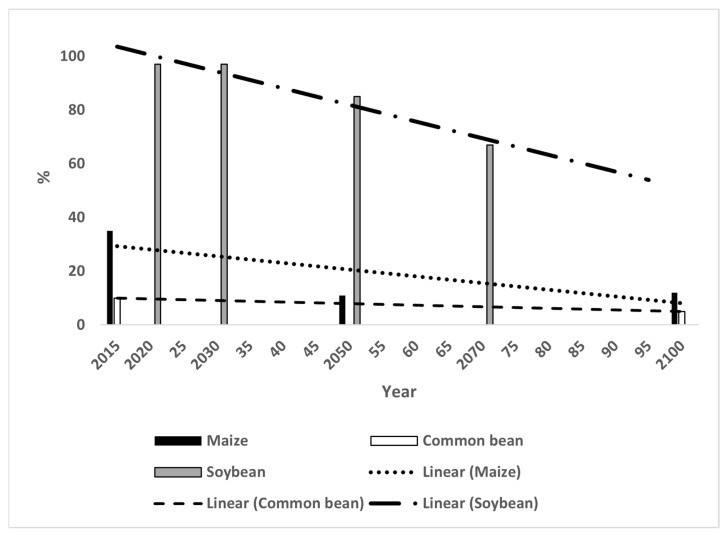
Weighted suitable climate for maize, the common bean and soybeans projected into the future for Colombia.

The incidences of bud rot disease were 20% and 90% and in 2015 and 2050, respectively. The death of OPs would take some years after infection to occur; hence, a figure for bud rot incidence in 2050 can be provided, despite the 100% mortality at that time. There would be no OPs in 2100 because of the mortality due to CC and BR. The WSC results were 35%, 11% and 11% in 2015, 2050 and 2100, respectively, for maize (Figure 2). For the common bean, the WSC results were 10% and 2% in 2015 and 2100, respectively. In the case of soybeans, the WSC values were 97%, 97%, 85% and 67% for 2020, 2030, 2050 and 2070, respectively. The trend line was from 108% in 2015 to 60% in 2100.

**Figure 3:**
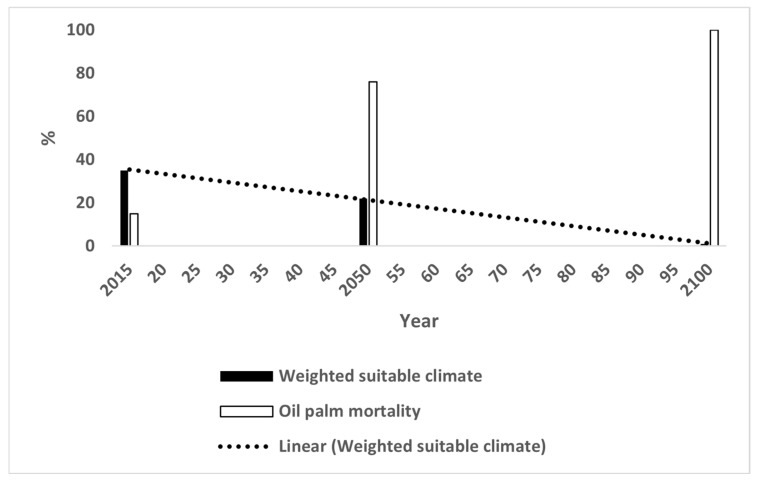
Weighted suitable climate and mortality from inclement climate and Fusarium wilt of oil palms projected into the future in Nigeria.

**Figure 4:**
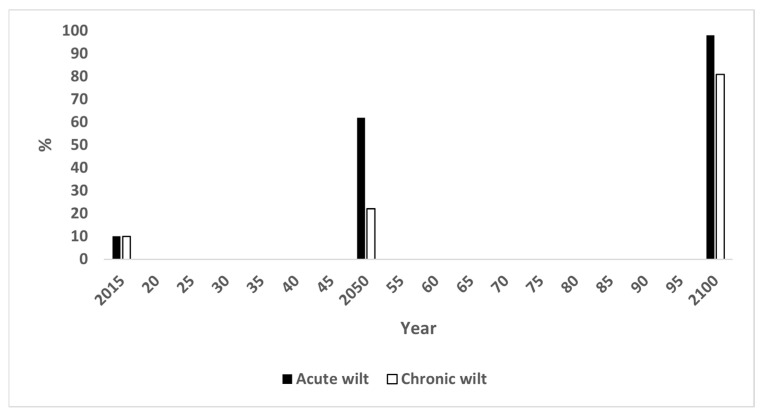
Acute and chronic Fusarium wilt of oil palms projected into the future for Nigeria. The oil palm mortality was 100% in 2100 (Figure 3). However, the actual death of the oil palms would take some years after that time to occur, and hence, incidences of Fusarium wilt are provided until 2100.

**Figure 5:**
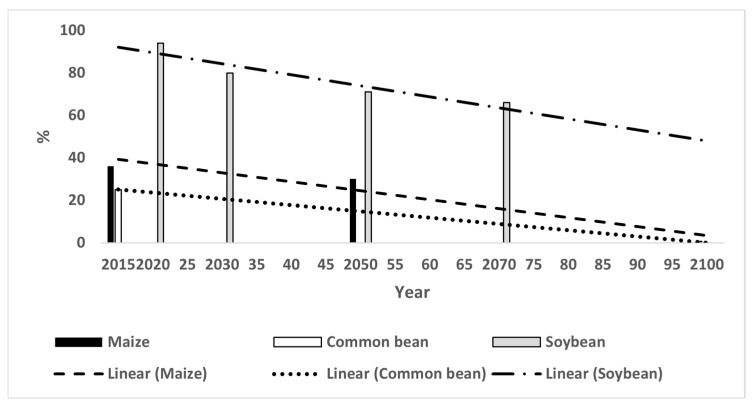
Weighted suitable climate for maize, the common bean and soybeans projected into the future for Nigeria.

The WSC data for OPs in Nigeria were 35%, 22% and 1% in 2015, 2050 and 2100, respectively, with a trend line of 36% in 2015 to 0% in 2100 (Figure 3). The mortality of OPs from the effects of CC and disease increased from 15% to 76% and then to 100% in 2015, 2050 and 2100, respectively. Incidences of acute Fusarium wilt disease were 10%, 62% and 98% in 2015, 2050 and 2100, respectively (Figure 4). The increases in chronic forms were determined to be 10%, 22% and 81% in 2105, 2050 and 2100, respectively. Figure 5 indicates the WSC results for maize, which were 36%, 30% and 0% by 2015, 2050 and 2100, respectively. The trend line was from 40% in 2015 to 4% in 2100. The WSC data for the common bean were 25% in 2015 and 1% in 2100, with the trend line having the same values. The WSC data for soybeans were much higher, at 94%, 80%, 71% and 66% in 2020, 2030, 2050, and 2070, respectively. The trend line was at 96% in 2015 and 50% in 2100.

**Figure 6:**
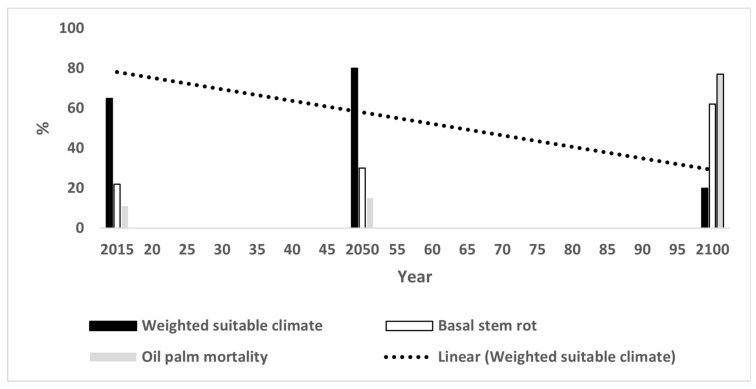
Weighted suitable climate, basal stem rot incidence and mortality from inclement climate and basal stem rot of oil palms projected into the future for Papua New Guinea.

**Figure 7:**
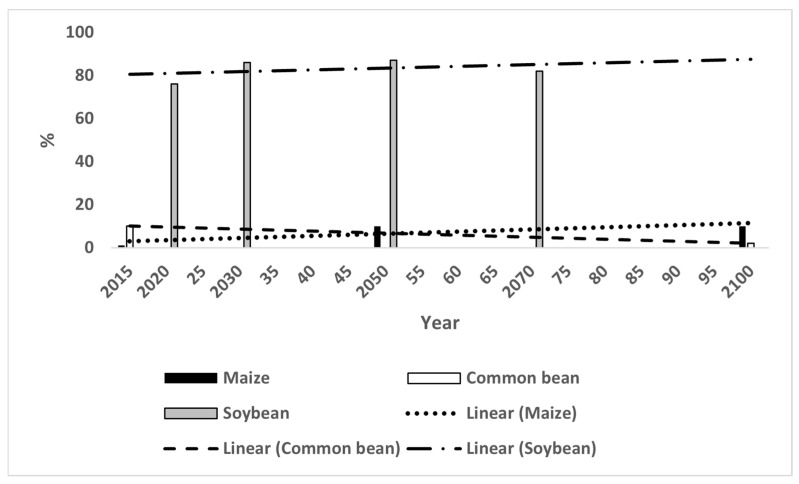
Weighted suitable climate for maize, the common bean and soybeans projected into the future for Papua New Guinea.

The WSC data for OPs in PNG are provided in Figure 6, with values of 65%, 80% and 20% in 2015, 2050 and 2100, respectively. The associated trend line was 79% in 2015 and 30% in 2100. The OP mortality from inclement climate and disease was 11%, 15% and 77% in 2015, 2050 and 2100, respectively. BSR incidences were 22%, 30% and 62% in 2105, 2050 and 2100, respectively (Figure 6). Maize had a somewhat increasing level of WSC, although from a low level (Figure 7). The data were 1%, 10% and 10% for 2015, 2050 and 2100, respectively. The trend line increased from 2% in 2015 to 11% in 2100. The levels for the common bean were 10% in 2015 and 0% for 2100. The WSC for soybeans remained at a high level throughout. The results were 76%, 86%, 87% and 82% for 2020, 2030, 2050 and 2070, respectively.

## 4. Discussion

OP disease incidences were projected as high in Colombia, Nigeria and PNG in the present paper, whereas those for introduced crops would likely be low (see Section 1 and below). The suitable climate for growing crops in the future was more favourable for soybeans than for the other crops, including OPs. Vegetable oil is an important common product obtained from OPs and most of the other crops considered herein, although other products are also obtained from these crops [6]. The situations for each country in terms of how OPs compare to the other crops are as follows. 

### 4.1. Colombia

The mortality of OP was 100% by 2050 when unsuitable climate and bud rot were considered (Figure 1). This is clearly an extreme value, and further modelling is required to test this scenario. In addition, in planta studies could be undertaken in the laboratory to determine the effect of possible future climate conditions on OPs and the likelihood of obtaining this degree of mortality. Nevertheless, the information indicates that action towards these ends is required.

The WSC data for soybean were much higher than those for OPs in Colombia from the present time to the common data point of 2050 (Figure 1 and Figure 2). The 2070 datum for soybeans was higher than the 2050 datum for OPs, indicating a greater resilience of soybeans to future climate conditions. These observations signify that soybeans are more sustainable than are OPs in Colombia and as such, they could be considered as a viable alternative crop in the future. The possible introduction of soybeans into areas where the crop has not been previously grown may result in lower incidences of diseases in general because they will be planted frequently and will not have endemic diseases established over long periods, as is the case with OPs. The crop may experience the “parasites lost” phenomenon, in which introduced plants tend to have fewer diseases, at least initially, as discussed in Section 1. Interestingly, modelling studies on the effect of future climate conditions on the diseases and pests of crops, including soybeans, indicate a reduction in these problems [30,31] which would be beneficial to the concept of replacing OPs with soybeans. The conditions for maize and the common bean do not offer sufficient advantages over those for OPs (Figure 2). An approximate 15% WSC result for rapeseed at the present time decreased to 10% in 2040–2059 [26], also indicating that this crop would not be a suitable replacement for OPs.

Corte’s-Cataño et al. [34] discussed the influence of climate on a variety of crops in Colombia, including OPs. The authors indicated that OPs would be affected positively by increases in temperature and stated that Paterson [35] determined that South America might maintain OP production, in contrast to the results for Southeast Asia. However, Paterson [35] did not state this, and the scenario for South America was one of a general decrease, with Colombia experiencing high OP mortalities in the future.

### 4.2. Nigeria

The WSC results for OPs was initially only 35%, although this level clearly maintains the current palm oil industry. This decreased further by 2050 and then drastically by 2100 (Figure 3). The high mortality value in Nigeria of 75% by 2050 for OPs due to Fusarium wilt and inclement climate reflects the sharp decline in suitable climate conditions and indicates that the industry would not be viable by this time (Figure 3). The WSC data for soybeans (Figure 5) were much higher than those for OPs by 2050, and soybeans could be substituted for OPs by this time. Maize and the common bean exhibited high percentages of suitable climate areas initially, and the level for maize remained moderately high by 2050. Maize has potential for complementing OPs, at least until 2050. The WSC data for the common bean and maize were at low levels by 2100, and the crops were not suitable replacements for OPs by this time. The future climate is clearly much more suitable for soybeans than for any of the other crops. There remained a large area of land suitable for growing soybeans even by 2070, the final year considered. The advantages over OPs achieved by planting soybeans, in terms of disease, has already been discussed for Colombia (see above). Finally, Nigeria showed no climate suitability for rapeseed at the present time, nor did it for 2040 to 2059 [26]. Rapeseed is not a suitable crop for Nigeria, according to this data.

Finally, Gloy et al. [35] refer to Paterson [36] as focusing on Nigeria in relation to OPs and claimed that shifts in climate suitability to the south and west of Nigeria were discussed. However, Nigeria was not the focus of Paterson [36], who was concerned with a much larger region of Africa, and the information in [35] is misleading.

### 4.3. Papua New Guinea

The WSC data for OPs in PNG appear distinctly different to those for Colombia and Nigeria, in that the suitability increased by 2050 (Figure 6). In addition, OP mortality rates from inclement climate and BSR were low. The 2100 WSC figure was low for OPs, contributing to a decreasing trend, and OP mortality reached an unsustainable level of 72% in PNG. On the other hand, a high percentage of WSC areas was maintained until 2070 for soybeans, which contributed to an increasing trend by 2100. The data showing the superiority of the soybeans over OPs were less convincing than those for Colombia and Nigeria by 2050, but were obvious for 2100. There was a high incidence of BSR in PNG, although less so than for the diseases of OPs in Colombia and Nigeria. In addition, BSR rates in PNG were determined previously as being lower than those for peninsular Malaysia and Sumatra, Indonesia, but higher than for Kalimantan [29]. Nevertheless, the BSR data reinforce the proposition of replacing OPs with soybeans because introduced soybeans are likely to have less disease (see above).

Only low levels of WSC for maize and the common bean were reported, and these crops do not represent suitable replacements for OPs. There was an approximate 15% WSC level for rapeseed at the present time, which changed to 0% in 2040 to 2059 [26]; thus, rapeseed would also not be a suitable replacement for OPs, on this basis.

Table 2, Table 3 and Table 4 indicate the potential for replacing OPs with soybeans from the perspective of climate suitability and OP mortality in Colombia, Nigeria and PNG. Importantly, the orientation of the suitable climate is also provided within the countries. The large reductions in suitable climate for OPs in 2050 in Colombia could be anticipated by planting soybeans after 2030. A large area from the west to the centre of the country will become particularly suitable for soybeans (Table 2). By 2070, there is a moderate decrease in climate suitability for soybeans in the north and northeast, making a shift to soybeans less favourable in these regions. There remains a strong case for planting soybeans when the data were extrapolated to 2100, although the north of the country may be less suitable for soybeans than the other areas.

In the case of Nigeria (Table 3), the status quo would apply before 2050. Soybeans could replace OPs by 2050 in the central and more northern parts of the country because the suitability for OPs decreases considerably in these regions. By 2100, soybeans could be planted in all areas to replace OPs, as there would be no OPs, according to the scenarios presented herein. The suitable climate for OPs increases by 2050 in PNG, and there would be a limited scope for replacing the palms with soybeans at that time (Table 4). The case for replacing OPs with soybeans is the strongest by 2100 because of the large decline in suitability for OPs at this time.

In general, the situation for Malaysia, Indonesia and Thailand also indicate that soybeans would be a suitable replacement for OPs, whereas maize and the common bean would not [6]. Rapeseed would also not be a suitable replacement in these countries, as discussed in Section 1.

Monocultures of crops are well known to have inherent sustainability problems deriving from disease and/or severe climate [6]. Furthermore, soybeans are straightforward to harvest compared to the requirements for OPs and can be mechanised more readily; the harvesting of OPs is labour intensive. These factors will be exacerbated by heat in the future, which will make manual labour more difficult or impossible [2,37].

A great deal of effort has been expended in attempting to control the diseases of OPs, but these have been unsuccessful for long-term control in plantations [38,39,40]. The diseases are likely to increase in virulence in the future, as discussed herein. A definite solution to the diseases would be to replace the OPs with another suitable crop not normally grown in the areas where OPs are currently grown. Soybeans may be such a crop. Introduced crops are likely to have fewer diseases, which has been described as the “parasites lost” phenomenon [9,41]. The introduced crop may develop more diseases in the longer term, but at least there would be an initial advantage. Existing studies that involve modelling the effects of the future climate on the diseases and pests of soybeans, indicate that the problems with soybeans will decrease [31], representing a distinct advantage over the scenario for OPs. Furthermore, in planta research could be undertaken to verify the disease levels of soybeans under CC parameters by challenging with soybean-associated disease and pests. The possibility of replacing OPs with soybeans represents a “cure” for the diseases. In addition, soybeans will be more resistant to disease per se compared to OPs because they will be growing in a suitable climate, unlike the situation for OPs. Vegetable oil will remain a major product of soybean production, which represents a positive outcome when palm oil can no longer be produced. This situation would also apply to Malaysia, Indonesia and Thailand, where soybeans could also replace OPs in some areas [6]. Other examples where alternative crops or livestock have been considered or introduced to cope with CC were discussed in Section 1.

Replacing oil palms with soybeans is not just a climate suitability issue. Factors such as yield, farmer adaptation, soil suitability, infrastructure, market economics, labour systems, and biodiversity impacts also require consideration before proposing large-scale crop replacement. Although replacing deteriorated OP plantations with soybeans may appear problematic, it is at least a potential option when compared to having no crop whatsoever.

## 5. Conclusions

The current paper compares the future climate models of four crops in three countries using the same modelling parameters. The narrative models for three diseases of OPs are also compared to the diseases of newly planted crops. Clearly, the future climate for soybeans is more suitable than that for OPs. Newly introduced soybeans may exhibit fewer diseases and will be replaced by disease-free plants, to some extent, after one season. Replacing highly diseased OPs with soybeans appears a valid course of action, based on the data provided in the current paper. Similarly, growing soybeans in regions where OPs cannot grow because of the inclement climate is also a possibility. As mentioned previously, there would be more factors to consider, other than future climate, if replacing OPs with soybeans was to become a serious proposition. On the other hand, it is fortunate that an alternative exists if the future climate affects OPs as severely as indicated herein and in reference [6]. Climate models are scenarios rather than predictions, presenting situations may reasonably occur in the future. Actions can be taken on the basis of the models, which can be modified as real time data emerges. These are highly significant recommendations for the OP industry that, together with the information in reference [6], are novel.

## Figures and Tables

**Table 1:** Mechanisms involved in the determinations of the various parameters employed in the current paper (values in percentages) for A., Colombia; B., Nigeria; and C., Papua New Guinea. The changes in the incidences of diseases and oil palm mortalities from those in references [27,28,29] were determined by estimating how the weighted suitable climate values in [27,28,29] differed from those in the current paper. WSC = weighted suitable climate.

A. Colombia							
				Time period			
*Parameter*		Current time		2050			2100
WSC [27]		70		50			2
WSC (Current paper)		70		61			11
Bud rot incidence [27]		20		100			100
Bud rot incidence (Current paper)		20		No OP			No OP
Bud rot mortality (Current paper)		20		No OP			No OP
Climate mortality [27]		0		20			No OP
Climate mortality (Current paper)		0		No OP			No OP
Total Mortality		20		No OP			No OP
**B. Nigeria**							
WSC [28]		35		25			4
WSC (Current paper)		35		22			1
Acute Fusarium wilt incidence [28]		10		60			100
Acute Fusarium wilt incidence (Current paper)		10		62			98
Acute Fusarium wilt mortality		10		62			96
Chronic Fusarium wilt incidence [28]		10		20			80
Chronic Fusarium wilt incidence (Current paper)		10		22			81
Chronic Fusarium wilt mortality		5		11			40
Climate mortality [28]		0		3			80
Climate mortality (current paper)		0		3			82
Total mortality (Current paper)		15		80			No OP
**C. Papua New Guinea**							
WSC [29]		65		75			22
WSC (Current paper)		65		80			20
BSR incidence [29]		22		32			60
BSR incidence (Current paper)		22		30			62
BSR incidence mortality (Current paper)		11		15			31
Climate mortality [29]		0		0			44
Climate mortality (Current paper)		0		0			46
Total mortality		11		15			77

**Table 2:** A qualitative comparison of the climate suitability of soybeans and oil palms in Colombia from the present time to 2070 and 2100 for soybeans [22] and oil palms [24], respectively. The orientations of the various climate suitabilities are provided to indicate approximately where soybeans could replace oil palms, according to the scenarios herein. An interpretation of the information is also provided. Abbreviations: HSC = highly suitable climate; SC = suitable climate; MC = marginal climate; UC = unsuitable climate; SB = soybeans; OPs = oil palm.

		Time				
		Present	2030	2050	2070	2100
Soybeans	Climate suitability	95% HSC; 3% SC; 2% UC in west.	Similar to present time.	North and north-east, SC; remainder, HSC.	Small parts of north and north-east, MC; no UC.	
Oil palms	Climate suitability	Large UC in west.		Much larger UC in west to middle than at present time.		Very little HSC; remainder, UC.
	Mortality	Containable; level of 20%		No OPs		No OPs
	Implications	Status quo	Anticipate 2050 with more SB planting.	All regions could be replaced with SB. North and northeast may be suboptimal for SB.	Confirms replacement with SB, but north and northeast only marginal for SB.	Probably still large areas for growing SB.

**Table 3:** A qualitative comparison of the climate suitability of soybeans and oil palms in Nigeria from the present time to 2070 and 2100 for soybeans [22] and oil palms [24], respectively. (Refer to the Table 2 legend for further information.)

		Time				
		Present	2030	2050	2070	2100
Soybeans		Increased SC in north; remainder HSC.	More SC in north.	Small MC in north; more SC overall; HSC in south.	Small MC in north; more SC and in south; small HSC.	
Oil palms	Climate suitability	HSC in south; small SC and MC in middle; remainder, UC.		Declined HSC in south; much more UC.		Almost all UC.
	Mortality	Containable level of 15%.		Serious mortality.		No oil palms.
	Implications	Status quo	Anticipate 2050 by beginning SB planting.	SBs to replace OPs in middle and north.	Anticipate total replacement of OPs.	Almost total replacement with SBs.

**Table 4:** A qualitative comparison of the climate suitability of soybeans and oil palms in Papua New Guinea from the present time to 2070 and 2100 for soybeans [22] and oil palms [24], respectively. (Refer to the Table 2 legend for further information.)

		Time				
		Present	2030	2050	2070	2100
Soybeans	Climate suitability	95% HSC; 5% UC in central west.	Similar to present time.	Similar to present time, plus minor SC in north and south.	Similar to present time, plus minor SC in south	
Oil palms	Climate suitability	HSC, with a central belt of UC.		More HSC, but similar to present time.		HSC deteriorates greatly; areas of MC in northwest; larger areas of UC centrally.
	Mortality	Containable level of 11%.		Containable level of 15%.		Unsustainable level of 72%.
	Implications	Status quo	Status quo	No reason to replace OPs, except in anticipation of 2100 climate.	Considerable potential to replace OPs for 2100 situation.	OPs could be replaced by SBs.

## Data Availability

The original contributions presented in this study are included in the article. Further inquiries can be directed to the corresponding author.

## Abbreviations

The following abbreviations are used in this manuscript:

BSR	Basal stem rot
OP	Oil palm
